# Shifting research culture to address the mismatch between where trials recruit and where populations with the most disease live: a qualitative study

**DOI:** 10.1186/s12874-021-01268-z

**Published:** 2021-04-21

**Authors:** Tanvi Rai, Sharon Dixon, Sue Ziebland

**Affiliations:** Nuffield Department of Primary Care Health Sciences, University of Oxford, Radcliffe Observatory Quarter, Woodstock Road, Oxford, OX2 6GG UK

**Keywords:** Clinical trials, Site selection, Research activity, Recruitment, Generalisability, Equity, Prevalence, Disease burden, Geographic variation

## Abstract

**Background:**

Research participation is beneficial to patients, clinicians and healthcare services. There is currently poor alignment between UK clinical research activity and local prevalence of disease. The National Institute of Health Research is keen to encourage chief investigators (CIs) to base their research activity in areas of high patient need, to support equity, efficiency and capacity building. We explored how CIs choose sites for their trials and suggest ways to encourage them to recruit from areas with the heaviest burden of disease.

**Methods:**

Qualitative, semi-structured telephone interviews with a purposive sample of 30 CIs of ongoing or recently completed multi-centre trials, all of which were funded by the UK National Institute of Health Research.

**Results:**

CIs want to deliver world-class trials to time and budget. Approaching newer, less research-active sites appears risky, potentially compromising trial success. CIs fear that funders may close the trial if recruitment (or retention) is low, with potential damage to their research reputation.

We consider what might support a shift in CI behaviour. The availability of ‘heat maps’ showing the disparity between disease prevalence and current research activity will help to inform site selection. Embedded qualitative research during trial set up and early, appropriate patient and public involvement and engagement can provide useful insights for a more nuanced and inclusive approach to recruitment. Public sector funders could request more granularity in recruitment reports and incentivise research activity in areas of greater patient need. Accounts from the few CIs who had ‘broken the mould’ suggest that nurturing new sites can be very successful in terms of efficient recruitment and retention.

**Conclusion:**

While improvements in equity and capacity building certainly matter to CIs, most are primarily motivated by their commitment to delivering successful trials. Highlighting the benefits to trial delivery is therefore likely to be the best way to encourage CIs to focus their research activity in areas of greatest need.

**Supplementary Information:**

The online version contains supplementary material available at 10.1186/s12874-021-01268-z.

## Background

It is well known that randomised controlled trials (RCTs) often lack adequate recruitment from the worst affected communities [1–6]. Relatively little is known about how RCT research teams make their decisions about where to recruit or of the factors that underpin their behaviour. The tendency to establish RCT recruitment in areas characterised by less disease appears intuitively contrary to the research teams interests. To address this imbalance in recruitment, we need to understand why current practice prevails. We draw on interviews with chief investigators in the UK, who since 2017 have been encouraged to focus recruitment for their National Institute of Health Research (NIHR) funded studies ‘*with and in the populations most affected’* [7].

Including the right locations and participants in trials matters for science, efficiency, equity, and research capacity-building. If trial participants do not represent the affected population, the findings may not be generalisable, compromising external validity. Clinicians may be unsure about whether and how to use findings that are based on participants who differ substantially from their own patients [8, 9]. In addition to regional variations in disease prevalence there is also variation in the incidence of disease risk factors, care pathways and patient outcomes, related to social deprivation and investment in clinical infrastructure. This is an issue of equity because participation in research benefits patients, [10] clinicians [11] and health care services [12].

**Fig. 1:**
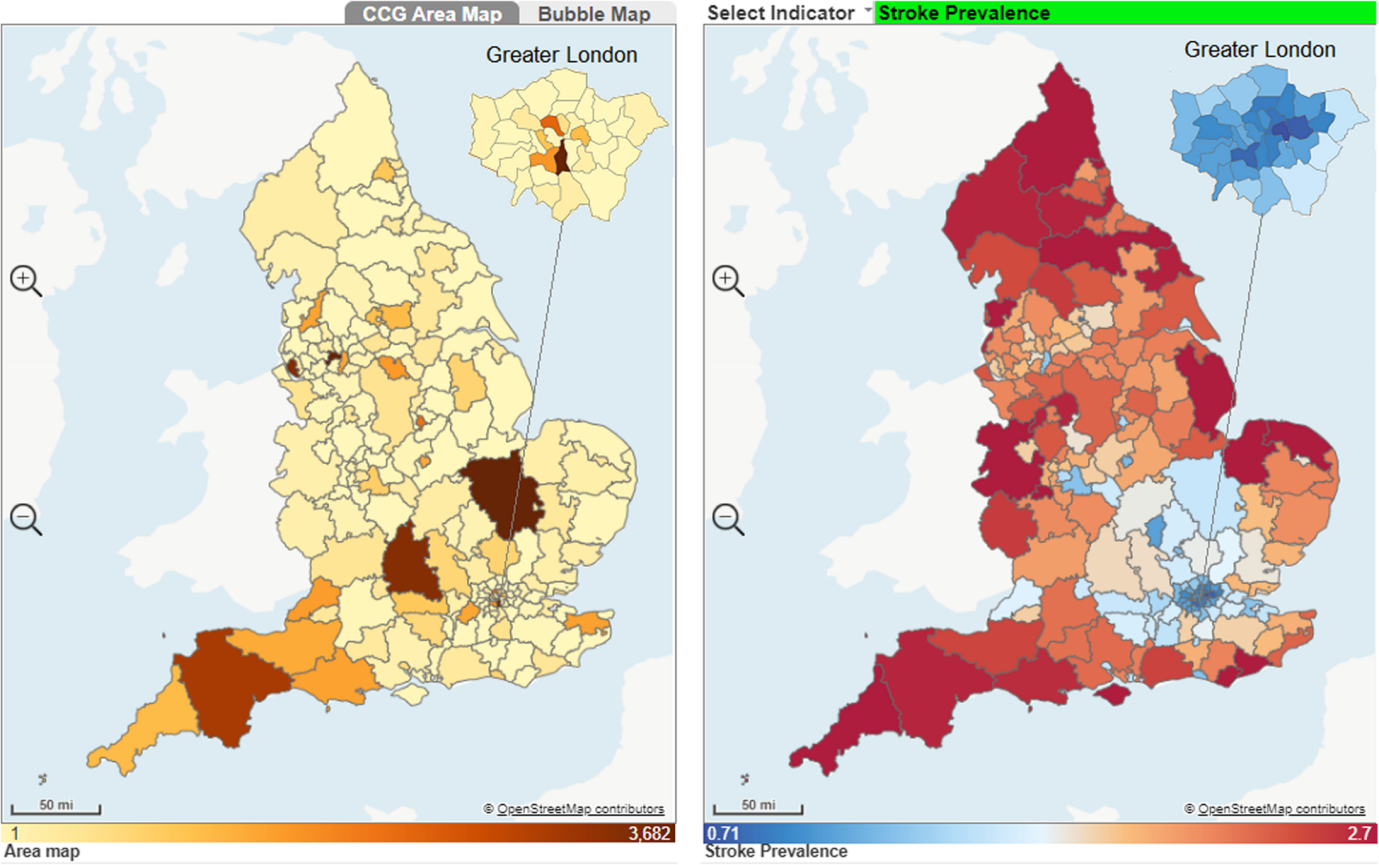
Heat maps for stroke in England: The map on the left shows NIHR CRN-supported research activity in England from April 2017 to March 2020 inclusive. The shading represents the number of participants recruited in each clinical commissioning group (CCG) area. Secondary care recruitment has been matched to CCG areas by looking up the site postcode against known postcodes within each CCG. The map on the right shows the percentage of patients with stroke or transient ischemic attack, as recorded on practice registers (percent). Data source: https://odp.nihr.ac.uk/

**Fig. 2:**
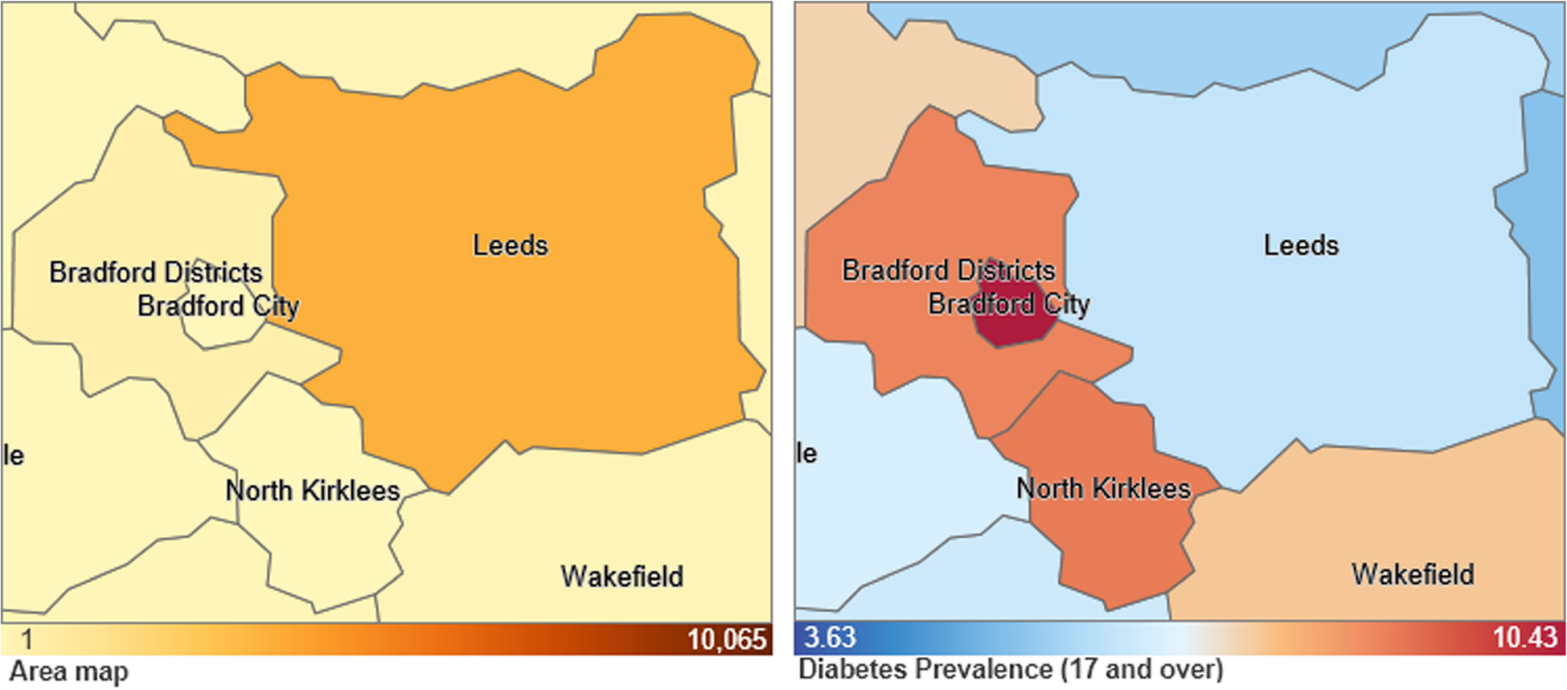
Heat maps for diabetes showing a close-up of Bradford and Leeds. The map on the left shows recruitment in NIHR CRN-supported diabetes studies in these areas from April 2017 to March 2020 inclusive, by CCG area. The map on the right shows the percentage of patients aged 17 and over with diabetes mellitus, as recorded on practice registers in 2016, by CCG. The prevalence data is from NHS Digital. Data source: https://odp.nihr.ac.uk/

In England, the NIHR Clinical Research Network (CRN) supports the planning, set-up and delivery of research. The CRN commissioned the development of “heat maps” [13] which compare the prevalence of specific conditions with the corresponding levels of current research activity across different regions in England (Figs. 1, 2). These maps show that some parts of the country with high prevalence of specific diseases recruit few research participants to trials relevant to those diseases, suggesting a need to build capacity in areas with low research activity. These data align with a more in-depth analysis that quantifies the extent of geographical mismatch between research recruitment and burden of disease [14].

One of the few studies investigating site selection behaviour [15] reported on a systematic review of NIHR-funded RCT protocols, a focus group and a survey of experienced RCT staff. They noted a discrepancy between what was considered important for “optimal practice” and what actually drives site selection. While generalisability of trial results in terms of population characteristics and clinical practice was desirable to trialists, the actual enrolment of sites was determined by more pragmatic considerations, such as convenience of location and where site staff are known to the chief investigator.

Here we report the findings from a qualitative interview study with a diverse sample of UK-based chief investigators (CIs) who have received NIHR funding for multi-centre RCTs. Drawing on their perspectives on how they make decisions about where to locate their multi-centre RCTs, we illuminate both why CIs choose to recruit where they do and what might be done to help shift current practice. This develops understanding about the processes that contribute to the observed misalignment between where the greatest patient need is and where research takes place. We are able to suggest potential mechanisms to shift behaviour towards greater involvement of sites that bear the heaviest burden of disease.

## Methods

We conducted semi-structured telephone interviews, between March–July 2019 with chief investigators (CIs) for NIHR funded multi-centre RCTs. The NIHR Clinical Research Network (CRN) provided an initial list of CIs holding grants for multi-centre (five or more sites), interventional RCTs that were either in progress or had completed since 2015. From this list we purposively sampled CIs based in different parts of the country, at different levels of seniority (although all were highly experienced researchers holding prestigious funding), across different specialities and interventions, and studying conditions known to be socially-patterned and those that are not. We included studies funded both before and after the 2017 NIHR letter from Professors Chris Whitty and Louise Wood encouraging the conduct of research where there is greatest health need [7].

Invitations, signed by (then) CRN CEO Dr. Jonathan Sheffield and Director of Industry Dr. Clare Morgan, were sent out by the CRN Co-ordinating Centre (CRNCC). CIs were invited to a 30-min telephone interview with an academic researcher. Invitations were sent out in waves, until we achieved the intended sample of 30 with appropriate variation among participants in terms of the aforementioned characteristics.

With input from the CRN, we created a topic guide for semi-structured telephone interviews (see Supplementary material). With participant consent, these interviews were recorded and transcribed verbatim. Interviews lasted between 20 and 90 min and were conducted by one of two university based qualitative researchers (TR and SD). Interviews resembled a guided conversation. Questions were open-ended and flexible to encourage participants to talk about whatever they deemed important to the topic of RCT site selection. The researchers referred to prompts and used follow up questions flexibly for further elaboration. We analysed the data using thematic methods [16], including a mind-mapping approach [17] which supports critical, reflective analysis. Mind-mapping is a way to organise and link related extracts from interview transcripts that have been grouped around themes, representing both the anticipated issues that we had set out to explore and those that were less expected and only emerged during the analysis. The OSOP mind mapping method [17] ensures that all of the relevant data are included in the thematic analysis, providing a robust and auditable approach. All three co-authors familiarised themselves with the transcripts and developed and discussed the mind-maps, initially in pairs and then together. Data files were managed using NVIVO v12 software.

## Results

### ‘Situation normal’

We asked the CIs about how they chose collaborating research sites for the multi-centred trials they led. Major considerations were the research track record of the collaborators, including well-established research and governance structures. CIs told us that when they were looking for new collaborators for their multi-site RCT they sought out enthusiastic local investigators who had previously delivered successful trials. Personal relationships and track records helped. These elements were often seen as critical to maintaining the high level of trust, communication and enthusiasm to make the trial a success. Notably, CIs researching rare diseases which might only be served by a few specialist centres nationally, described having only a limited pool of potential sites to choose from.

*We knew people in all of, we could have got (many) sites because we knew all the clinicians and surgeons working in various hospitals, so it was a matter of asking people whether they were interested in the study. [ … ] It was based on past research, and also, on reputation and people who you knew would probably be able to deliver with respect to pushing through recruitment. (CI 4)*

Clinical researchers often have senior mentors within their field. Mentorship may reinforce conservative considerations about what is important in site selection, for instance by favouring research sites that offer a ‘safe pair of hands’ and ‘trusted contacts’.

*So, I think the majority of the times, it’s the old boys network and who people know [ … ], so if I was doing another trial now, I’ve obviously got loads of experience of who can deliver and who can’t and we definitely would use that knowledge to work out who to invite to enter into another trial (CI 28).*

This status quo is strongly reinforced by concern that if recruitment and retention lag behind expectations, funders might close the trial, which in turn reinforces risk aversion when choosing sites. CIs are very conscious of the need to cultivate their own reputation as a successful triallist.

Sites that are relatively new to research were often perceived as lacking infrastructure and requiring extra resources and time to set up. Several CIs described these as risky ‘research naïve’ sites, likely to involve a lot of effort for little return, delays in recruitment or loss to follow-up and potential close down of the site. Some CIs described using a “one strike and you’re out” approach (CI 20), whereby if a given site failed to deliver, they would not be asked again, or would go to the bottom of the “pecking order” for subsequent trials (CI 25). Reputations stuck and sometimes meant that a site would not even be considered if they were not expected to be able to deliver.

**Fig. 3:**
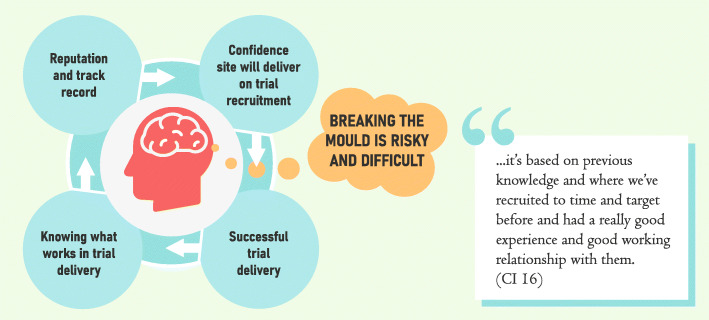
Breaking the mould is not easy for chief investigators of large trials. Source: Image created by the authors. Copyright owned by Nuffield Department of Primary Care Health Sciences, University of Oxford

**Fig. 4:**
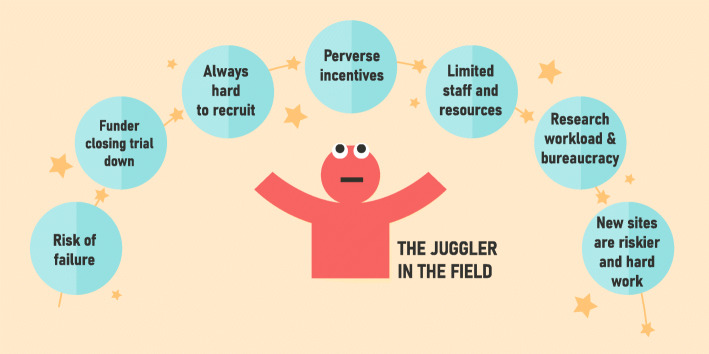
Chief investigators of multi-centre trials juggle a lot of priorities. Source: Image created by the authors. Copyright owned by Nuffield Department of Primary Care Health Sciences, University of Oxford

As Figs. 3 and 4 illustrate, breaking the mould is not easy, with CIs juggling a lot of different priorities.

### Shifting the culture of site selection

We now turn to what might help shift this ‘situation normal’. When we asked CIs for their views about research being conducted in areas of greatest health need, there was a broad consensus that arguments for equity and capacity building were persuasive, but practical obstacles were identified that could intervene against this. Our analysis of the interviews suggests that to be effective attempts to change the culture of site selection could harness the strong motivations that CIs have to complete their multi-centre RCTs to target. We identified six candidate approaches to encourage this shift, considering researchers, funders and infrastructure support. These are illustrated with anonymised extracts from the interviews.

#### Actively partner, nurture and trouble shoot in new high prevalence sites

Sites that are relatively new to research may require nurturing through partnerships and extra support to deliver trials. This could include tailoring and preparing local governance applications and preparing supporting materials to suit local needs as well as assistance in data management. CIs who nurtured new sites and remained responsive to their needs throughout the trial, reported that these sites were often enthusiastic and performed well.

*[It was] a small District General Hospital that doesn’t take part in many studies … they were really enthusiastic because we sent them everything they needed. So, you know, all the participant information sheets we sort of localised for them. We sent […] everything they needed. They didn’t have to generate worksheets. They didn’t have to generate their own localised PIS [participant information sheets] because they’re not really experienced in doing these things, so we did everything for them and that’s what made the difference because they realised we were willing to put ourselves out for them. We made it easy for them. (CI 12)*

The above quote shows how early support in the form of study materials tailored to them, enabled a less research-savvy site to come on board with enthusiasm.

*I don’t believe in adding more sites. I think you, that’s a bit of a default response to poor recruitment, open more sites. Where actually you need to work out what’s going in the sites, I think. That’s where we tend to put our energy if there’s a problem. We don’t open more sites, we try to help the open sites to recruit better. You just end up going to the scene of the crime and finding out what they’re really doing. (CI 28)*

Instead of looking to add more sites when recruitment falters, this latter quote illustrates a potentially more sustainable approach, whereby the trial team develops a site’s capacity to conduct research.

#### Work with appropriate patient and public advisers in setting up the study

In England, all publicly-funded research needs to demonstrate appropriate patient and public involvement and engagement (PPIE). As the following quote illustrates, the identification of potential PPIE contributors needs to be considered and planned carefully.

*… patient representatives who often sit on the national committees, [ …] are very educated, very selected people and sometimes what we need is to have feedback from what I call ‘real life’ patients. [ …] We want to hear from, you know, the average X patient who’s often from deprived areas, you know, doesn’t always have great family support and so on and that is difficult to access. (CI 13)*

Ensuring that the PPIE contribution is relevant to the research setting, research question, and to populations most commonly affected by the condition may require accessing people who are seldom heard in research [18]. This may require extra effort (e.g. travel, training costs, working outside standard working hours) as well as consultation with community representatives and area specialists who can give pragmatic advice about aiding the appropriate PPIE contributors to come on board [19, 20].

#### Consider conducting qualitative research during study set up

Embedded qualitative research within large trials has become more common and can illuminate how the different stages of an RCT are enacted in practice. Examining the detail of how study participants are invited to participate in research can provide insights to support recruitment and retention. (see QuinteT approach [21]) This can be particularly valuable in supporting and developing recruitment in new sites.

*we always use [ …] qualitative research investigation and we always request resources to be able to do that because that examines the, the recruiter’s perception, the patient perception and it improves, you know, you’re able to identify what these barriers are and then you’re able to fix them and you improve recruitment dramatically [ …] the interviews go to social medicine experts who will listen to the tapes, write transcripts, find out where the barriers are. Analyse them, come back to the recruiter, discuss with them how they talk to the patient. Whether they are sources of bias that could be eliminated. A very, very elaborate piece of work, but actually it makes or breaks recruitment. (CI 18)*

#### Optimise use of research infrastructure

The CRN in England has a key role in developing and supporting new research sites. For example, their heat map data on disease prevalence and research activity [13] can be used to guide recruitment and site selection. Not all of the CIs we spoke to were aware of the breadth of CRN activities. Awareness, and use of, infrastructure support is not evenly distributed. New sites are likely to need guidance about what support is on offer.

*the sites, actually they were not aware that they could apply for resource from the local CRN, [ … ]. So, we actually had a, I don’t know if you call them roadshows, … … .you know, just to inform the potential sites of the facilities that are out there in the local network. As I say, if they could ask for additional resource, research resource particularly if obviously they were recruiting well. [ …] So, so a lot of it is actually about just organisation and management and being aware that [ …] there is the NIHR and there are research networks in each region and also informing them about their, certainly the [Speciality] CRN lead for that region who can help them with regards to any additional resource. (CI 29)*

Another CI suggested that incentives could be used to support research activity in previously under-researched geographical locations, perhaps including ‘double points’ for recruitment.

*I would differentially reward recruitment in those areas, so rather than having one portfolio point per recruiter whatever it is, if it really is the North East who’s got the highest incidence and we’re recruiting there, it’s not going to be, I kind of feel they should get, you know, double the points, if you like, at least to a certain period of time until they get to a point where they’ve established how to do it. (CI 28)*

Targeted investment, support and mentorship programmes for new researchers in areas of greatest need could increase capacity and perhaps slow the tendency for promising researchers to move to more established centres to pursue their careers. CIs pointed out that high disease prevalence often corresponds with high clinical workload and staff pressure, challenging attempts to get involved in research. Protecting clinician time and embedding research into clinical roles could support developing research capacity in these areas. This support may need to be provided equitably, rather than equally, so that highly clinically active sites can access research support that is proportionate to their needs.

*Undoubtedly, you know, consultants need that protected time and if you don’t get it, particularly in busy DGHs (district general hospitals), the consultants just won’t be, won’t be able to engage in research. They’ll be pulled into clinical work, so funding for consultant time is, I think is paramount.” (CI 25)*

#### Funder’s role in setting expectations

While not all research funders have a strategic interest in encouraging the research activity they fund to follow patient need, they are in a strong position to stimulate change by setting specialised goals and rewards in ways that promote this. Some funders (usually those in the public or voluntary sector) may choose to target investment towards regional capacity building. For example, established research teams can be incentivised to work with less experienced colleagues in areas of highest patient need (see for example [22]). Although forced ‘*marriages of convenience’* were not popular, some CIs suggested that investment in ‘*key people on the ground whose mandate is to collaborate’* (CI 23) could lead to gains for efficiency and capacity building.

When funders maintain a very tight focus on recruitment numbers CIs suggested this could lead to perverse incentives. If an ‘*over-focus on counting*’ (CI 27) adds urgency to the recruitment process this could detract from taking the time to nurture and build research capacity in newer sites. Likewise, CIs may hesitate to stray beyond known territory if they feel it could potentially compromise their trial timelines and personal reputation (thus reinforcing the ‘situation normal’ described above). If funders required research teams to provide breakdowns of recruitment by, for example, geographic, socio-economic and ethnic groups this might involve more work but, as one CI noted:

*if there’s an imperative and it’s kind of insisted on by NIHR, people will budget accordingly and they will make allowances accordingly. But otherwise, I suspect most people will start with what they know and the places that they know” (CI 21)*

#### Publicise examples of successful recruitment in new research sites in areas of higher patient need

We spoke to a few CIs who had successfully recruited in newer sites characterised by high disease burden. They had come to see such sites as fertile grounds for recruitment, providing a pool of patients who are not over-researched, and local clinical research teams who can focus more fully on the trial. One CI suggested that close knit communities, often with wide experience of the health condition being studied, may engender strong feelings of altruism and a willing pool of participants.

*“You speak to the patients and they’re all dead keen to take part, you know, because something needs to be done if it’s to help somebody else and there’s a lot of altruism going on with people from [location], you know, there’s a huge community spirit amongst these areas of deprivation.” CI 12*

These CIs contrasted the experience above with working in highly research-active sites where several RCTs may be recruiting simultaneously, competing for patients and leading to *‘research fatigue’* for staff and patients. Concerns regarding the extra time and resources involved in supporting newer sites may need to be tempered by a consideration of the many likely rewards from including patient communities that shoulder the heaviest burden of disease, and therefore have a rich and diverse experience of the condition, as well as potential gain from trial participation.

## Discussion

### Summary of main findings

Our study illuminates why the ‘situation normal’ in recruitment has persisted. CIs are concerned that trying to recruit and retain trial participants in less known, less research-active sites is risky and could undermine their trial’s success. This concern is sharpened by the fear that funders may close the trial if recruitment (or retention) is low, with potential damage to the CIs’ research reputation. Many CIs recognise that recruiting trial populations which reflect the geographical distribution of disease burden would increase the external validity of their trial results. While there was no demur about the importance of equity and capacity building, there is little doubt that the CIs we talked to were most strongly motivated by the need to deliver good science, with their RCTs completing on time and to target. Support and incentives to access and develop new sites and populations is likely to be the most promising and sustainable way to encourage CIs to recruit where this is greatest patient need.

Accounts from those CIs who ‘broke the mould’ suggest that actively identifying and nurturing new sites could be more successful than repeatedly returning to familiar (highly research-active) sites. If publicised, these CI experiences could encourage others to follow a similar path, especially if funders provide targeted support to research teams. Where ‘heat maps’ are available, showing disease prevalence and research activity, will help CIs to plan site selection. Qualitative research and early, appropriate PPIE can provide useful insights for a more nuanced and inclusive approach towards recruitment. Research funders might avoid perverse incentives by requesting more granularity in recruitment reports.

### Strengths and limitations

This is the first study where a purposive sample of CIs of multi-centre RCTs have been interviewed about how they choose their research sites. Our participants showed great interest in the topic and often talked for well beyond the 30 min we had requested. We only included NIHR funded multi-centre RCTs conducted in the UK and all had access to CRN support. Our findings may not transfer to international settings with different research infrastructure or different geographical and social patterning of disease.

### Implications of our findings

Bower et al. [14] recently explored geographic variation in research recruitment activity by prevalence of long-term conditions in England. Similar to the heat maps in Fig. 1, they found disproportionately low recruitment activity in areas with higher prevalence of long-term conditions, particularly diabetes and mental health conditions. They highlight the need for indicators to assess the fit between research and need, as well to increase understanding about how better alignment could be achieved.

The mismatch between what clinicians and patients want and what researchers choose to investigate has been noted [23] and this mismatch motivated the Priority Setting Partnerships of the James Lind Alliance [24] whereby patients help decide what topics or questions should be prioritised for research. This approach may need to be extended to ensure representation from under-researched regions. PPI advocates, such as INVOLVE in the UK, recommend research be co-produced with patients [25]. The growing popularity of community-based participatory approaches [26, 27] supports long-term academic-and-community partnerships for which the community stakeholders are strategically identified from areas of greatest patient need. Pre-trial qualitative research with staff involved in delivering or recruiting to a trial can help to refine the design and delivery of trials [28]. This approach could be valuable for identifying the specific resources newer sites might need for successful recruitment and retention of participants. In addition to practical challenges such as adequate staffing, organisational capacity and completing regulatory procedures, there is also the initial and ongoing work of building and supporting a research culture, that may involve engaging clinicians who are not directly involved in the trial [29]. Poor communication between CIs, local investigators and recruitment staff can lead to misunderstandings about the trial rationale, including concepts such as “equipoise”, and this can be detrimental not only to recruitment but also to interpretation of the study findings [30].

The nature in which the Covid-19 epidemic spread and cases surged across England provides yet another explicit illustration of the geographic unevenness in the distribution of disease, with populations in areas of economic deprivation often suffering a greater disease burden [31]. Different regional populations also vary by ethnic diversity, population density, types of housing and public facilities, and exposure to pollution which can have important effects on health, and may need to be factored into trial site selection. Concerns about “waste in research” were raised more than 10 years ago [32] and still remain a problem [33]. The most common reason for discontinuation of trials is reported to be due to poor recruitment [34]. The careful inclusion of research sites with high disease prevalence has the potential to overcome both these problems, as long as there is a realistic assessment and planning for the support needs and costs for achieving this [23].

## Conclusions

**Fig. 5:**
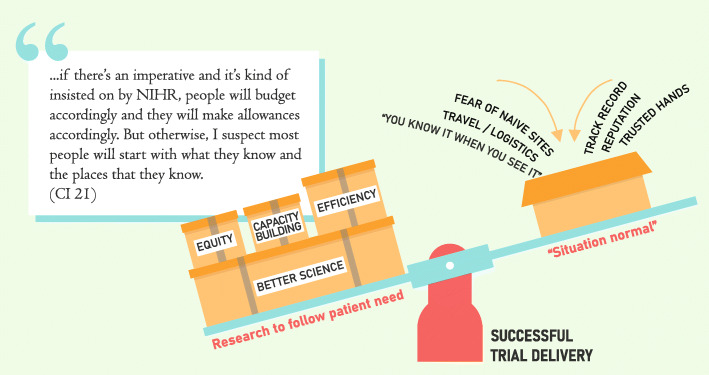
The factors that can help tip the balance so that research follows patient need. Source: Image created by the authors. Copyright owned by Nuffield Department of Primary Care Health Sciences, University of Oxford

While public funders of research, such as the NIHR, are committed to the values of equity, efficiency, capacity building and the production of robust science, we found that it is the latter that is likely to be the main driver for CIs when making decisions about research sites (see Fig. 5). CIs want to conduct multi-centre RCTs that have strong internal and external validity and are delivered to time and budget. Demonstrating that they can achieve this by focussing their research activity in areas of greatest need will support the desired cultural shift in researcher behaviour. While improvements in equity, efficiency and capacity building are likely to be the welcome attendants of such a shift we conclude that CIs are primarily motivated by their commitment to delivering successful trials.

## Supplementary Information

**Additional file 1.** Topic guide CI project. The topic guide for the semi-structured interviews conducted.

## Data Availability

The interviews collected and analysed for this study are not publicly available as they contain confidential and identifiable information. Please contact the corresponding author for more detail.

## Abbreviations

CEO: Chief Executive Officer
CI: Chief investigator
CIs: Chief investigators
CRN: Clinical Research Network
CRNCC: Clinical Research Network Co-ordinating Centre
DGH: District General Hospital
NIHR: National Institute of Health Research
OSOP: One sheet of paper
PPIE: Patient and public involvement and engagement
PIS: Participant Information sheet
RCT: Randomised Controlled Trials
UK: United Kingdom
