# Supplementary material for: Shifting research culture to address the mismatch between where trials recruit and where populations with the most disease live: a qualitative study
Source: BMC Med Res Methodol. 2021 Apr 21;21:80. doi: 10.1186/s12874-021-01268-z (PMC8058580; doi:10.1186/s12874-021-01268-z)
Supplement: Supplementary file 1 — Additional file 1. Topic guide CI project. The topic guide for the semi-structured interviews conducted. [file 12874_2021_1268_MOESM1_ESM.docx]

**Draft Topic Guide for NIHR CRN project.**

Thank you for agreeing to participate in this interview on behalf of the NIHR, conducted by me at the University of Oxford. You have been asked to talk to us because you are named as CI on an (index project) NIHR multi-site RCT <ADD NAME OF PROJECT EACH TIME>. We will work through a series of questions to gain greater insight for NIHR to better understand how Chief Investigators make decisions about which sites to include in multi-centre Randomised Controlled Trials (RCTs).

In order to save me writing down what you’re saying and avoid recall problems, I am going to audio record this call – is that okay? We will be writing a report and might want to use some quotes from our interview. These will be anonymised and non-attributable. Will that be okay? (Unless you wish to have your name attributed).

1. Could you start by telling me briefly about how the idea for this study (the multi-site RCT mentioned above) came about?
   - How many study recruitment sites are involved?
   - Were these specific study recruitment sites always planned or have they changed (if so, any reasons)?
   - How did you select your study recruitment sites?
   - What factors influenced the choice? *(prompts: existing collaborations/ people you had worked with before, availability of specific facilities, prevalence of condition in area, recommended as having the capacity and capability to deliver, the site team made a direct approach etc.)*
   - Looking back from where you are now, is there anything you would have done differently?
   - Did you receive support or guidance from the NIHR funder or the NIHR Clinical Research Network in selecting and working with research sites?
     - Did you find that helpful?
     - Was there anything that you feel would have been useful?
     - Was there anything that you feel could have been improved?
2. I would like to ask you now about your wider experience/ thoughts about the set-up and delivery of multi-centre RCTs, as either a chief or co-investigator or principal investigator (this could include studies funded by other agencies, i.e. not NIHR-funded studies).

- In your experience, what influences which study recruitment sites are selected to deliver multi-site RCTs?
- What do you consider important/priority factors in influencing this decision?
- What are the challenges when selecting and setting up study recruitment sites for multi-centre RCTs?
- Have you experienced changes in study recruitment sites during the course of (other) RCTs? *(prompts – where more sites added/ did you have to close non-recruiting sites and open others, could site not open due to local issues)*
  - If so, for what reasons did this happen?

1. The NIHR is taking seriously the issue of research taking place in areas of need. You may be aware of a letter written by Chris Whitty and Louise Wood from the NIHR (DHSC) in 2017, highlighting NIHR should conducting research with the populations affected by particular health problems. In some geographical areas there is relatively little research taking place.

- What are your perceptions about the usefulness of conducting research in areas of greatest need?
  - Advantages
  - Disadvantages
  - Challenges
- Do you think this 2017 letter from Whitty and Wood has had any impact, either personally or on the clinical on research community in selection of study recruitment sites?
- Do you know of any examples where disease prevalence has been factored into your own or colleagues’ work?
- Are there things that you think the NIHR could do to support research teams in optimising site selection for multi-centre RCTs?
  - *(Note first allow time for their thoughts, if needed could use examples following as a prompt for discussion. For example with practical aspects, such as a site identification service, with data about prevalence, sites which have recruited to similar studies, specialist facilities or recruitment rates; with support for sites less experienced at recruiting to research studies etc)*
- Is there anything else you think it might be useful for us to consider about the practice and logistics of multi-centre RCT site selection, or that you haven’t had a chance to mention yet?

END Thanks etc
